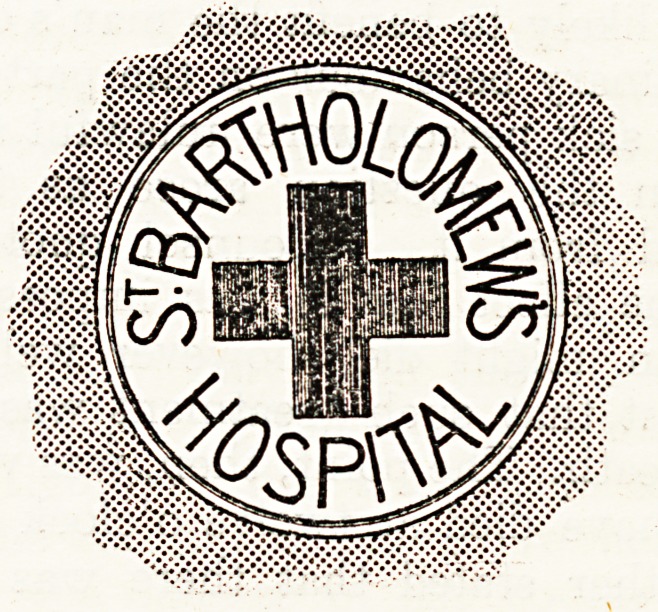# Hospital and Institutional News

**Published:** 1915-07-24

**Authors:** 


### HOSPITAL AND INSTITUTIONAL NEWS.

### HOSPITALS AND THE SPIRIT DUTIES: THE EFFECT OF THE ACT.

The Finance Bill has now passed into law without embodying any provision for the relief of hospitals from the heavy burdens imposed by the spirit tax. In respect of this tax, therefore, hospitals are in much the same position as they were, except that they will have to comply with the new conditions arising out of the Immature Spirits (Restriction) Act, in order to obtain repayment of the surtax on rectified spirit. On the report stage of the Finance Bill Sir Henry Craik formally moved the insertion of a clause similar to that brought forward by the Government on the Committee stage, but consented to its withdrawal on receiving from the Chancellor of the Exchequer a repetition of an assurance he had previously given to Sir Philip Magnus that he agreed in principle to a grant from public funds to be based on the duty paid on spirits by hospitals last year, subject to investigation of an Advisory Committee working in connection with the Board of Customs and Excise. This should be as acceptable to hospitals as the original proposal, but unfortunately there is no guarantee that time will be found at an early date for Parliament to give effect to the undertaking. As all students of Parliamentary procedure are aware, there is all the difference in the world between an expression of approval of a principle and the embodiment of that principle in an Act of Parliament; and although Mr. McKenna's intentions are good, many things might happen in these days of quick changes to prevent him from carrying them out. It is also important to note that Mr. McKenna said the undertaking was given subject to the agreement of all parties, and that the moment he had said this a representative of the temperance party said he did not think the principle was accepted by all parties. Clearly, therefore, it would have been in the interest of hospitals to have inserted Mr. Bridgeman's clause in the Finance Bill; a bird in the hand is worth two in the bush. The British Medical Association has been successful in preventing hospitals from obtaining the concession originally offered, but is in accord with the principle of a grant to hospitals; the only way in which the Association can atone for the wrong it has done is by utilising its efforts unsparingly in order to ensure that the principle of the grant is speedily endorsed by Parliament. [For correspondence see page 359.]

### THE BRITISH HOSPITALS ASSOCIATION: CONVERSATION ON JULY 30.

An important meeting of the British Hospitals Association is to be held at Charing Cross Hospital on Friday, July 30, at 3 P.M., when Mr. J. Courtney Buchanan, Secretary of the Metropolitan Hospital, will open a Conversation on "The Shortage of Medical Officers in the Voluntary Hospitals." The chair will be taken by Mr. H. Wade Deacon, Chairman of the Liverpool Royal Infirmary, and those expected to take part in the proceedings include Sir Cooper Perry, M.D., Medical Superintendent Guy's Hospital; Dr. T. Jenner Verrall, and Dr. Alfred Cox, the Medical Secretary of the British Medical Association. Readers of The Hospital will recall that the annual conference of the British Hospitals Association was to take place this year at Liverpool, but in view of the present situation, and the difficulties thereby placed in the way of hospital managers, many of whom in consequence would not be able to leave their work to take part in such proceedings, the idea of a conference has been regretfully abandoned. It must not be supposed, however, that all such activity is to cease. In fact, the Council of the British Hospitals Association is now considering what policy would best secure the interests of the voluntary hospitals during a year in which a formal conference would be impracticable. An announcement may be expected at the forthcoming Conversation, and, indeed, means may be found at the Conversation itself, at all events to touch on the no less pressing question of duty-free spirit for hospitals. If this can be arranged in such a way as to prevent the discussion from dissipating itself on diverse issues the plan should prove welcome. In any case full advantage should be taken by members of the opportunity afforded them of reviewing the whole situation created by the shortage of medical officers, which is likely in October to press so hardly upon those voluntary hospitals, the great majority, to which no medical schools are attached.

### THE WAGES OF AMBULANCE DRIVERS.

The London County Council is experiencing considerable difficulty in recruiting, at the present rate of wages—namely, 35s. a week—suitable drivers and attendants for the ambulance stations which will be ready to begin work in the near future. It has also been found that, as regards drivers, men have taken early opportunity of leaving the Council's service, presumably with the object of bettering their positions. As regards attendants, it was hoped to be able to obtain the services of men holding first-aid certificates with experience of ambulance work, and of good judgment and general knowledge. But it has been found that the wage of 35s. a week does not attract sufficient men of the right stamp. It has, indeed, become evident that if suitable drivers and attendants are to be secured, wages of not less than 38s. a week, with £5 bonus at the end of each six months of satisfactory service, should be paid. The Special Committee point out that it is essential that the interests of the Council should be thoroughly safeguarded by the engagement of none but competent employees for work of such a special character as this. It has accordingly been decided to grant this wage and gratuity.

### THE WAR'S EFFECT ON THE HEALTH OF SCHOOL CHILDREN.

More than one school medical officer has been enabled to record, since the outbreak of the war, an improvement in the general nutrition of the children in his schools. The suggestion is obviously that the Government allowances to dependants of soldiers, and the great decrease of unemployment, have afforded parents an opportunity to provide a more liberal dietary for their offspring. Possibly, also, there has been in some districts a more generous allowance of tissue-building foods in the free meals given at schools. But the home diet is probably the factor which has undergone most improvement in consequence of the greater spending power as regards food of the more conscientious class of parent. In the annual report of the medical officer of health for Salford, there are given figures which bear out the foregoing, and, in addition, there is recorded a fall in the infantile mortality rate. Seeing the improved conditions referred to above can only have exerted an influence during the last four months of 1914, there is given some grounds for hoping that the reports for this current year, when completed, will show a substantial reduction of the infantile mortality rate and a higher standard of nutrition among school-children. This result, if realised, would be some return to the nation for the immense sums which are being distributed to the dependants of our fighting men.

### RELATION OF SCHOOL DOCTOR TO TEACHER: A REGRETTABLE INCIDENT.

The increasing interest and influence of school teachers in the work of medical inspection has been appreciated by medical officers in all parts of the country, and the assistance which teachers have rendered in bringing parents into closer touch with the school doctors has been of great value. Our readers will recall a recent article in The Hospital for June, in which the experiences of a school doctor in connection with the giving of popular lectures to parents were recorded. It is, therefore, with some surprise that an instance must be recorded in which the managers of a school appear more ready to "crab" the efforts of the school doctor than to encourage him to seek the co-operation of the teachers. At a recent meeting of the Cambridgeshire Education Committee it was reported by the School Attendance Sub-Committee that communications had been received from the Rev. Canon Evans, regarding some inspection at a provided school. It was represented more particularly that "all attempts to give directions of any kind to teachers otherwise than through the managers must cease," that notice was to be given of *all* visits of the school medical staff, and that no arrangements affecting any person in the school were to be made by the school medical officer without full information being given beforehand to the managers. As any school medical officer would at once admit, to carry out such "orders" would interfere seriously with the work of school medical inspection, for occasions are easily conceivable when it would be contrary to the interests of the work to give notice of visits for re-inspection, and for the control of infectious disease. It should have been obvious to the managers that for the school doctor to give directions to teachers in the indirect manner suggested by Canon Evans, would be both undesirable and impracticable. Most of the communications made in that manner would lose force, miscarry, or be misinterpreted. After the Clerk of the Council had pointed out to the sub-committee the duties of school managers, the Rev. Canon Evans received a reply in which the freedom of the school medical officer to act unhampered in regard to the above-mentioned points was asserted on behalf of the Education Committee, subject, of course, to notice in the case of routine inspections in the usual way.

### A LESSON FOR CAMBRIDGE SCHOOL MANAGERS.

Such disputes are nowadays exceedingly rare and it seems a great pity that in this instance things could not have been amicably arranged before attaining the indignity of an official disputation. It is interesting to note that in the last report of the deputy school medical officer for Hertfordshire, great emphasis is laid on the value and importance of the part played by the school teacher as an agent in school health. Special mention is made of the influence which teachers are enabled to exercise in promoting a higher standard of personal cleanliness among the children, particularly among girls, though boys also are successfully stirred up. Outbreaks of infectious disease, too, have been prevented or minimised before now, by the observation and immediate action of school teachers, who, now realising more fully their responsibilities, are appreciating and encouraged by the recognition that their co-operation is receiving.

### SIR FREDERICK TREVES.

The health of Sir Frederick Treves was made the subject of a question in the House of Commons last week. The questioner was Mr. W. Thorne, Labour member for the South Division of West Ham, who asked the Under-Secretary of State for War whether he was aware that Sir Frederick Treves had acquired a complaint in Alexandria which had got gradually worse; whether he would state the nature of the complaint; whether Sir Frederick had been inoculated with any vaccine or serum previous to its occurrence; and whether he would state with what vaccine or serum or other such preparation he was inoculated. Mr. Tennant's answer was as follows: "I have no information on this matter, and if I had I doubt whether I should be justified in making it public." As it is obvious that the question had in view larger matters than its important personal interest, it is well to put it on record as an instance of the keenness with which the progress of inoculation is being followed at the present time.

### THE EMPIRE HOSPITAL—FOR OFFICERS.

Arrangements have now been completed whereby forty-two rooms in the Empire Hospital, Vincent Square, Westminster, have been taken over by the War Office as an extension of Lord Knutsford's scheme for the special treatment of officers suffering from traumatic neurosis. The hospital, we understand, is to be managed, subject to the control of the Director of the Army Medical Service London District, by a joint committee composed of members of the board of directors of the Empire Hospital and of the committee of the Special Hospital for Officers, under the chairmanship of Mr. Herbert Samuelson, who is the chairman of the board of the Vincent Square institution. While the hospital in Palace Green is devoted more especially to cases of functional nervous disorders arising from shock, it is intended to accommodate in the Empire Hospital cases of organic nerve injury—that is to say, cases suffering from physical wounds requiring the aid of nerve specialists.

### SECRETARY OF STOCKPORT INFIRMARY RESIGNS.

UNUSUAL interest was given to the monthly meeting of Stockport Infirmary last week, when the following letter was read from Major Tyler: "I beg to tender one month's notice for the termina-

T. Everitt Innes] MAJOR CYRIL TYLER. [Stockport.

tion of my appointment as secretary of the Stockport Infirmary, which I have held for twenty-one years." Mr. E. F. Ward's speech admirably outlined Major Tyler's connection with the institution, when, regretfully moving the acceptance of the resignation, he reminded the meeting that this July it was precisely twenty-one years since Major Tyler became assistant secretary. A few months later he became the active colleague of the hon. secretary, Col. Wilkinson, at whose death, in 1900, Major Tyler took sole control of the secretarial department. The chairman of the medical board, Dr. Hyde Marriott, said that Major Tyler had been more than a secretary, and, like the late Col. Wilkinson, had brought a strong personality to bear on the work. Dr. Marriott, by the way, has a record of practically the same length as Major Tyler's in connection with Stockport Infirmary. Punctuality, method, fairness, and character were the qualities alluded to by Dr. R. A. Murray in a eulogistic speech. Discipline and accuracy were as necessary in an institution as in the Army, and Dr. Murray concluded by proposing that an address recording his services and an honorarium of 100 guineas be presented to Major Tyler. In seconding this Mr. H. P. Guy paid valuable tribute to the voluntary system by saying "there must be something of the human interest and value in an institution of this kind when we find a man like Major Tyler devoting himself wholeheartedly to it." The treasurer, Colonel Sykes, remarked that part of the secretarial work had been to act as assistant to the treasurer and from the time that he, the speaker, succeeded his father in that post, Major Tyler had been a valuable help to him. A tribute was also paid to the retiring secretary's work by Mr. J. H. Thorpe, on behalf of the clergy.

### MAJOR TYLER AND THE VACANCY AT STOCKPORT.

THE above account of Major Tyler's happy relations with his colleagues at Stockport would be incomplete were not a brief reference made to the military career which preceded his appointment as a hospital secretary in 1894. He became attached to the Indian Army and reached Bombay in 1859, but his first important experience of active service occurred eight years later, when he was appointed assistant to the Director of Land Transport in connection with the Abyssinian Expedition. Lord Napier of Magdala accorded recognition to his services, and in 1877 these were considered sufficiently valuable for the Chief Director of Transport, Kandahar, to ask for, and for the Commander-in-Chief, Bombay, to decline to part with them. On his retirement he was granted a special pension in consideration of his long and meritorious service, and, as stated above, in 1894 he succeeded Mr. John Andrew in the secretarial department, where Mr. Andrew himself had been at work for twenty-two years. Indeed, long records of service of its secretaries are remarkable and characteristic of Stockport Infirmary, since the late Colonel Wilkinson, alluded to above, was honorary secretary for no fewer than fifty-two years. It remains only to add that Major Tyler has been appointed a life vice-president of the hospital, and will spend his well-earned leisure at Southport, removed, that is to say, from those temptations to work which his friends might expect to prove irresistible to his energies did he spend his retirement at Stockport itself. In inviting applications for a successor to the post of secretary thus rendered vacant, the hospital stewards place the age-limit of applicants at fifty-five years, make it a whole-time appointment, and offer a salary of £160, rising by annual increments of £10 to £200.

### SAVING ON THE TUBERCULOSIS SERVICE.

THE London County Council has already taken in hand the matter of economising, and has issued instructions to its committees to consider where reductions in estimates may be made. The Public Health Committee, after full investigation, report that there will be a saving in connection with the treatment of tuberculosis, but they are unable to suggest a reduction in the votes in respect of other services. Difficulty has been experienced in securing residential accommodation for tuberculous persons, owing to a very large number of beds being reserved for wounded and sick soldiers; but, by a fortunate circumstance, the number of adult tuberculous patients requiring treatment in hospitals and sanatoria has also been smaller than was expected. The expenditure under this head will, therefore, be less than anticipated, and it is proposed to reduce the vote by £6,000. Taking into account the Government grant which would have been receivable if this amount had been expended, the net reduction will be £3,000. The contributions towards the expenditure of Borough Councils in tuberculosis dispensaries schemes will also be smaller in the aggregate than was estimated, and the saving under this head will probably amount to £1,000. Is it worth while?

### PROFESSOR DENYS OF LOUVAIN ON TUBERCULOSIS.

An instructive address on tuberculosis and tuberculosis work was delivered by Professor Denys of Louvain at the sixteenth annual general meeting of the National Association for the Prevention of Consumption, held on Thursday last week at 20 Hanover Square, W. Professor Denys summed up the measures to be adopted in protecting the human being from tuberculosis in two recommendations—namely, the use of the sputum flask by patients, and the sterilising of milk. He discussed the low infectivity of tubercle and submitted experimental evidence in favour of the immunity of the contact from infection when the simple precaution of collecting and destroying the expectoration is carried out. There is great need for the public mind to grasp the basic truth that tuberculosis is a disease of relatively low infectivity which may readily be controlled. The alarm with which cases of closed tuberculosis are viewed in certain districts is the cause of much annoyance and inconvenience to the patient. The closed case, if diagnosed and treated as one of tuberculosis, is branded as such, while the open case, if it goes by any other name, is accepted as innocuous. Professor Denys' concise statements with regard to the infectivity of tuberculosis and the vulnerability of the tubercle bacillus outside the human body are welcome. Any danger which may exist in connection with an open case of tuberculosis lies in the fact that the precautions are so simple that they are liable to be overlooked, and that these precautions can be carried out by the patient himself. The moral is for all county tuberculosis officers and their assistants to use their best endeavours, not only with insured patients, but with their *neighbours* also, to drive home these facts, so that open cases may not be treated by "friends" as pariahs, and suspected cases may not be concealed for fear of social ostracism.

### POOR-LAW INFIRMARIES AND COAL CONTRACTS.

With the coming approach of winter many Boards of Guardians are going into the question of the supply of coal for their infirmaries and other institutions. The Contracts Committee of the St. Pancras Guardians, in recommending the acceptance of certain tenders for coal, have indicated to the board that the expenditure on heating is likely to be greatly increased for the coming winter on account of the increased prices required by contractors. The price required for "house coal"—which previously was about 20s. per ton—is now 30s., whilst Welsh steam coal for boilers is 39s. per ton, a considerable rise in price as compared with those in previous years. We understand that the Guardians have stipulated that, should Government action result in a fall of prices, the contractors must reduce their prices accordingly. The St. Pancras North Infirmary is to provide increased storage accommodation for a reserve stock of coal. The matter of buying in winter stocks of coal for our hospitals at the present time is a difficult problem; where contracts are being fixed, some such arrangement for a reduction in the event of a fall in prices caused by Government action would appear to be a useful stipulation, although, as terms of contracts vary, other provisoes may commend themselves elsewhere.

### DISPENSARIES AND THE INCIDENCE OF TUBERCULOSIS.

It is gratifying to note that, with the exception of four Metropolitan Borough Councils, the establishment of tuberculosis dispensaries is now a fact throughout London. The laggards ere this will have been probably reduced to three. In the majority of cases the Borough Councils have taken advantage of existing dispensaries, either those connected with some hospital where the disease has been made a speciality, or with a voluntary agency which has carried on the work prior to the introduction of the National Insurance Act. In these instances the records of cases that have been treated in the past should be of great value in dealing with cases arising in the same family or locality. As to what cause the apparent decrease in the number of tuberculosis cases is to be attributed, opinions vary. Those upholding the National Insurance Act ascribe the good results to this measure, whilst others credit them to better classification, which has reduced the number of "complaints" formerly ascribed to tuberculosis by a less hasty diagnosis. At all events, an apparent and marked decrease in the number of tuberculosis cases remains. What the beneficent cause may be we invite our readers, in the light of their experience, to suggest.

### "PLACES OF SAFETY" UNDER THE MENTAL DEFICIENCY ACT.

The London County Council have addressed a further letter to the Southwark Board of Guardians with reference to the action of the former authority in sending to the Newington institution a mentally deficient patient from Balham, some considerable distance away and in the area of the Wandsworth

Board of Guardians, which was referred to in The Hospital, page 305. The Asylums and Mental Deficiency Committee, whilst recognising the right of the Southwark Board of Guardians to send only patients from their own parish to their institutions, point out that the number of available "places of safety" south of the Thames is very limited. It would be a great convenience, they add, therefore if the Southwark Guardians would agree to receive in the Newington institution, if accommodation were available under the provisions of Section 15 of the Act, defectives other than those from Southwark. The cost would have to be borne by the neighbouring authorities. The Guardians realise that both in Lambeth and Wandsworth there is much more spare accommodation than at the Newington institution, and that Boards of Guardians there should be called upon to take care of their own defectives, and not saddle them on to obliging neighbours. It is doubtful, however, if the Southwark Board of Guardians would see the Central Committee in a difficulty. With that readiness that has often characterised them they would perhaps permit their institutions to be used pending such time as other and more equitable arrangements could be made.

### INSURANCE PRACTITIONERS AND THE WAR.

At a meeting of the London Insurance Committee, held on July 22, the Medical Benefit Sub-Committee presented a report dealing with the urgent question of the treatment of insured persons during the absence of medical practitioners on active service. This report is in support of a scheme prepared by the Panel Committee, which provides that the Committee shall undertake that the services of a panel doctor shall be available for any insured person entitled to obtain treatment from a practitioner on the London panel who is absent from his practice because he has received a commission in his Majesty's Military or Naval Forces. The adoption of the scheme depends upon the consent of the Insurance Commissioners to such an amendment of the Medical Benefit Regulations as would have the effect of suspending during the period of the war the right of an insured person on the list of a practitioner engaged on active war service to change his doctor at the end of the year following the conclusion of the war, or the practitioner's retirement from such service, whichever is the earlier. It is proposed to ask the Commissioners to make the necessary alterations in the Regulations. In times of peace insured persons would probably object to the temporary abrogation of their rights in this respect, but under the present abnormal conditions it is hoped that they would consent to waive their rights if by doing so they could release for service with the Forces a number of panel doctors.

### THE "BART.'S" HOSPITAL BADGE.

To our article and illustrations of War Service Badges for Hospital Staffs may now be added that granted at St. Bartholomew's Hospital, which we also illustrate this week. The adoption of this badge was largely due to the initiative of the Medical School authorities, who started it for the benefit of their students, who had expressed a need in this respect. It has also been given to the hospital clerks. The Medical School is responsible for the cost in both cases. In addition to this another section of the hospital's employees—namely, the labourers and others—are anxious to be provided with a badge, and, it is understood, one of bronze, but not necessarily with the same design and inscription, is in contemplation. In view, however, of the main question of the desirability or otherwise of granting badges, on which more than one opinion prevails, it is interesting to quote in this connection a passage from a letter of Sir George Pragnell, which appeared in the *Times* of Saturday last, under the title of the "Truth about Recruiting."

> Another question which might be settled with advantage is that of badges. Too many badges have been instituted already, and the cry is still for more. Take the vexed question of "Badges for the Unfit." Who are unfit? And isn't it enough for patriotic, sensitive, and disappointed men to learn (probably for the first time) that they are defective without being compelled to proclaim the fact? But I maintain that the majority of them ought not to be rejected at all! There is some service they might well render. Take, for example, only the case of clerks. Thousands of strong, healthy men are wasted in the Army on clerical and detail work which could be done as well, or better, by trained clerks who have been refused because they are not strong enough to spend a winter in the trenches. Every large employer can point to splendid fellows in his counting-house who have scarcely had a day's illness in their lives, but who have been rejected by an Army doctor. Business men could find a place for practically every volunteer, and I look forward to one badge only being allowed—apart from the King's uniform. It should be for all men and women between the ages of sixteen and sixty who place themselves at the disposal of the Government for any service anywhere until Prussia is "beaten to her knees."

Sir George's above remarks on the badge question will interest our readers. How far is he right?

### SERIOUS CHARGE AGAINST A DOCTOR.

Dr. Samuel Percy Johnson was lately charged on remand at Birmingham Police Court with causing the death of a man, concerning whom the Coroner's jury had returned a verdict of "Death from natural causes." Counsel for the prosecution declared the circumstances to be very unusual and extraordinary. They were there, he added, not to inquire into the cause of death, but into the guilt, if any, of the accused. He put two points to the

Court. First, whether a man was guilty of manslaughter who, whilst doing an unlawful act, caused the death of another. From this two questions arose: Was the doctor doing an unlawful act, and, if so, did the death result from it? He went on to say that the doctor's behaviour was extraordinary, because, evidently, in attacking a man whom he knew to be *in articulo mortis*—hitting him twice with a cane, and thumping him on the side of the neck—the doctor was not carrying out a treatment likely to benefit the man's disease. If this extraordinary behaviour on the part of a medical man to a sick person were unlawful assault and battery, then the question arose as to whether death ensued from it. Counsel quoted medical opinion to the effect that it was impossible to say when a man might die, however well or ill he might be, but that such treatment was calculated to expedite death. The point, then, was whether the man would have died at twelve o'clock that night. Counsel further stated that there was no ill-will between the deceased and the accused, except that the doctor had informed the deceased's wife that he had slapped her husband's face that morning "just to rally him round." Apparently an argument was proceeding in the street before the assault occurred.

### WHY THE CHARGE WAS DISMISSED.

On behalf of the accused, counsel replied that there was no evidence on which a jury could convict his client of any unlawful act, and certainly not of an act which had accelerated the death of the deceased. Indeed, the doctor had shown great kindness to the deceased, and had treated him without fees. The deceased went to see Dr. Johnson, and waited outside his house, where the doctor saw him on leaving his residence to fulfil a social engagement. The doctor offered to talk to the deceased as they walked along the road; while they did so, the man showed signs of faintness, and the doctor told him to pull himself together and to wait for a tram. The Magistrate said that he did not think any jury would convict on the evidence presented. Counsel for the prosecution said that, in that case, he should offer no evidence. Dr. Johnson was thereupon discharged.

### THE WAR AND THE SITE OF THE NEW BRADFORD ROYAL INFIRMARY.

Highfield House, on the estate which is to be the site of the New Royal Infirmary at Bradford, is being used as an auxiliary military hospital, where there are fifty beds. At Bowling Park, the Epileptic Colony, eighty beds have been given, and at the Royal Eye and Ear Hospital twenty-three. At Woodlands, the Bradford Royal Infirmary's Convalescent Home, there are soldiers from Leeds and Halifax, in addition to those sent from Bradford; altogether three hundred and seventy-seven have spent their convalescence there. The first men arrived after the retreat from Mons. From that day onward many who have taken part in famous engagements have come to Bradford wounded and very tired, and have gone away with renewed strength and courage.

### AN ARMY PHARMACEUTICAL SERVICE.

The British Pharmaceutical Conference, which usually lasts for four or five days and is accompanied by a round of festivities, compressed its annual meeting this year into less than two hours. Instead of being held at Scarborough, it took place in London, at the headquarters of the Pharmaceutical Society, on July 14. The President, Major E. S. Peck, took as the subject of his address, "Pharmacy Organisation and the War," and outlined a scheme for the reorganisation of the Pharmaceutical Service of the Army. The two branches of the proposed scheme included, first: The supply, collecting, and storage of the necessary drugs and dressings; and, secondly, the provision of trained men to supervise this supply and its distribution to the various units in the base and field hospitals. It would, he thought, be wise for the State to have in peace-time a separate organisation for the production as well as for the collection and storage of medical necessaries, capable of expansion in wartime, so as to enable them to supply at once a proportion at least of the quantities required. By this means there would also be in continuous training in Army methods a body of men who in a sudden emergency could be easily augmented. He outlined the duties of pharmacists in the central store, in the base hospitals, and field hospitals, and there will be general agreement with his suggestion that, considering there are twenty-five hospitals with an average of 500 to 1,500 beds, at least a trained and qualified pharmacist should be in authority at the head of each of these institutions.

### THIS WEEK'S DRUG MARKET.

Business in the drug market continues active; this is mainly the result of the continued demand for drugs for the Medical Services of the Allies at this usually quiet season. Changes in values have not been numerous since the last report was written, but the general tendency of prices is still in an upward direction. The high quotations for mercurials are well maintained in consequence of the strong tone of the quicksilver market, and a further advance is quite probable. Little business has been done in cod-liver oil at the fancy prices quoted, and as events are moving it is at least possible that these extreme values will not be maintained; the attempt on the part of Norwegian dealers to form a combine has not been wholly successful, and so long as some of the larger firms continue to hold aloof the attempt to force up prices to figures altogether disproportionate to the statistical position of the market can hardly be expected to succeed; viewing the situation from all aspects prospective buyers would be wise to delay their purchases. High prices are still asked for bromides, which continue scarce. Sulphonal is dearer. Phenazone and phenolphthalein are somewhat lower in price. The price of cocaine is not quite so firmly maintained. Carbolic acid has a tendency to advance in value. There is again a fair demand for quinine, and the price has an upward tendency. Hyposulphite of soda has a lower tendency in price, further supplies being expected.

## Figures and Tables

**Figure f1:**
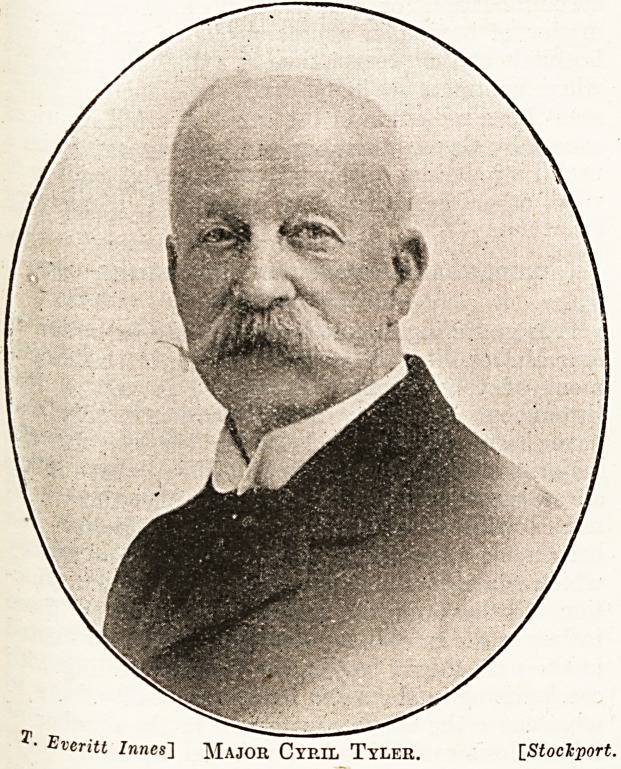


**Figure f2:**